# Supplementary figures and images for: A sensitive tissue factor activity assay determined by an optimized thrombin generation method
Source: PLoS One. 2023 Jul 19;18(7):e0288918. doi: 10.1371/journal.pone.0288918 (PMC10355404; doi:10.1371/journal.pone.0288918)

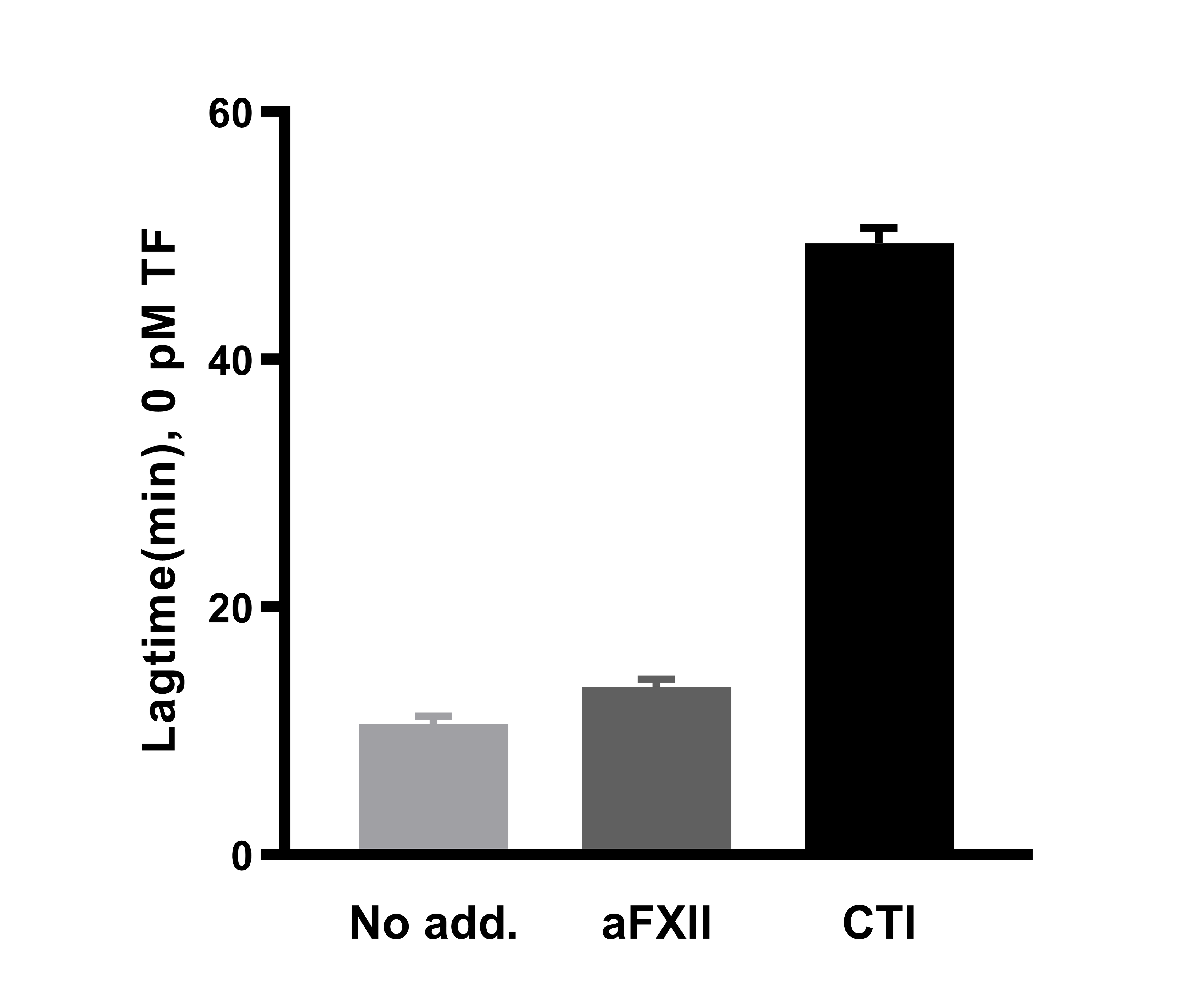

Supplement: S1 Fig — Addition of a FXII increases LT by some minutes, but addition of CTI to the blood increases LT substantially. The bars show mean and SD of 4 plasma samples. (TIF) [file pone.0288918.s001.tif]

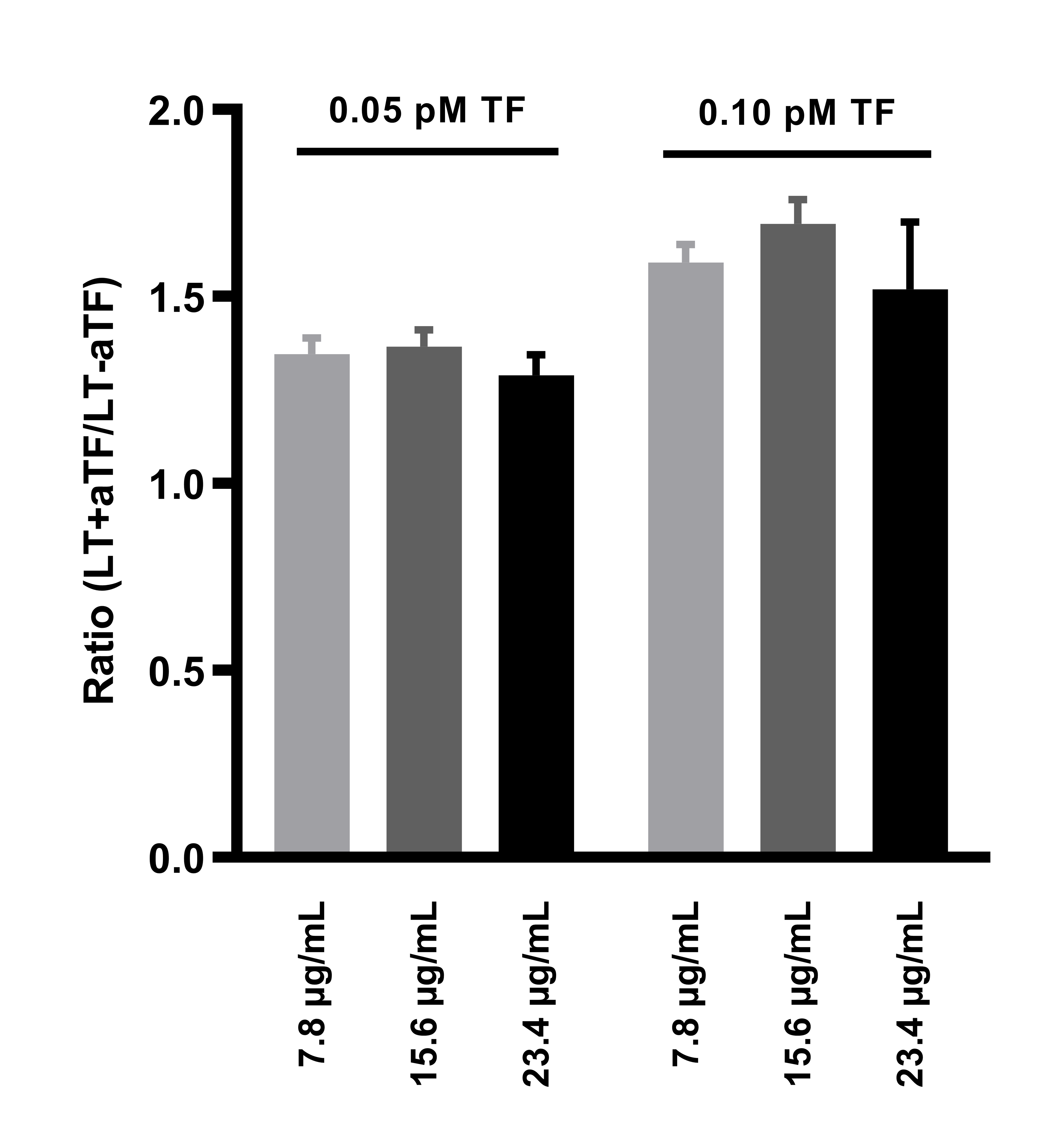

Supplement: S2 Fig — The figure shows the ratio between LT in the presence and the absence of antibodies, when TG is activated by 0.05 and 0.1 pM TF. I.e., the more the antibodies inhibit TF the higher ratio will be expected. It appears that increasing aTF to higher concentrations do not increase the ratio indicating that 7.8 μg/mL is sufficient to inhibit the TF activity. The bars show mean and SD of 4 plasma samples. (TIF) [file pone.0288918.s002.tif]

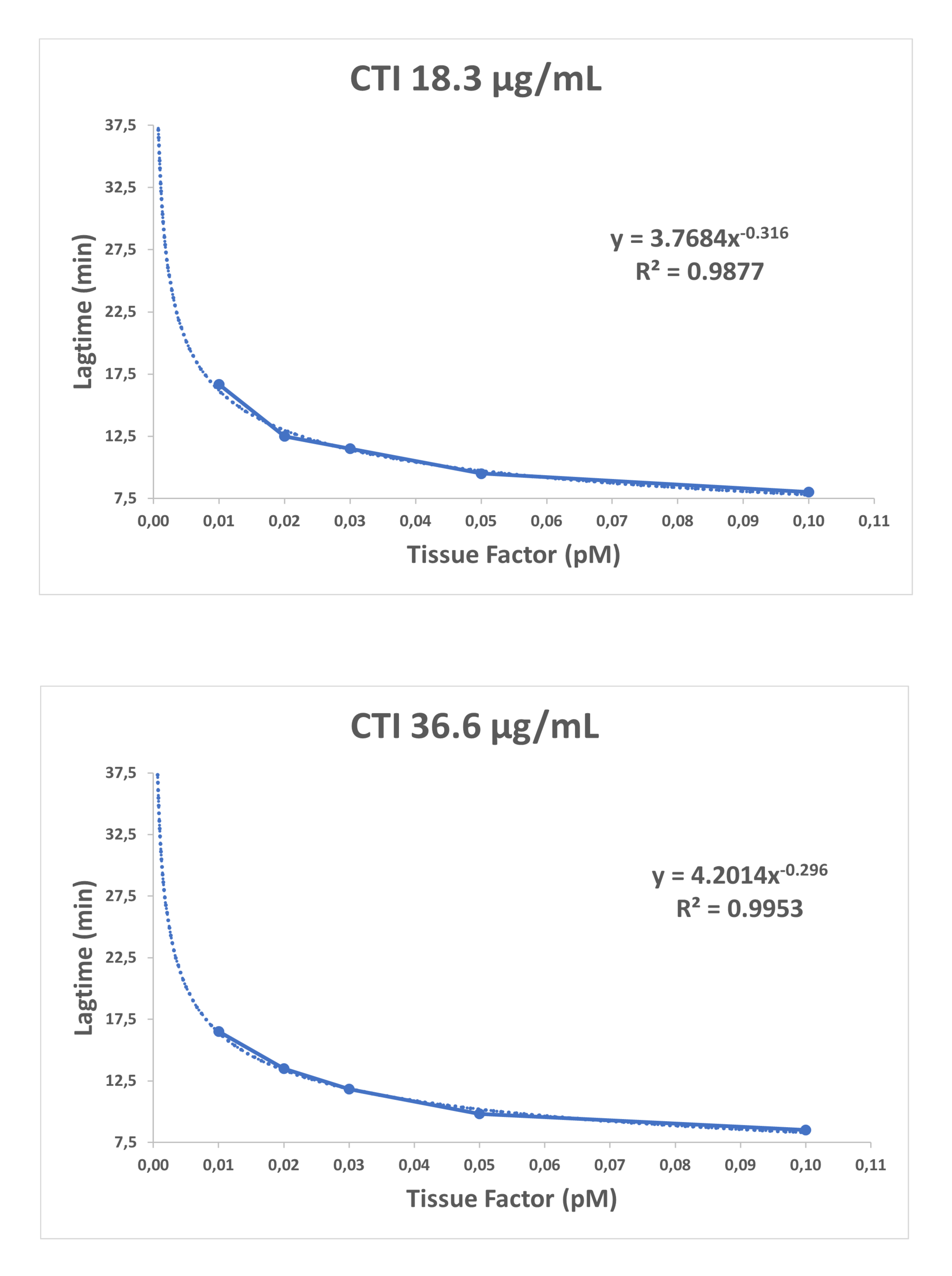

Supplement: S3 Fig — Calibration curves (i.e. LT vs TF concentration) using 0.01–0.10 pM TF to plasma from blood samples where CTI was added in the two concentrations. (TIF) [file pone.0288918.s003.tif]

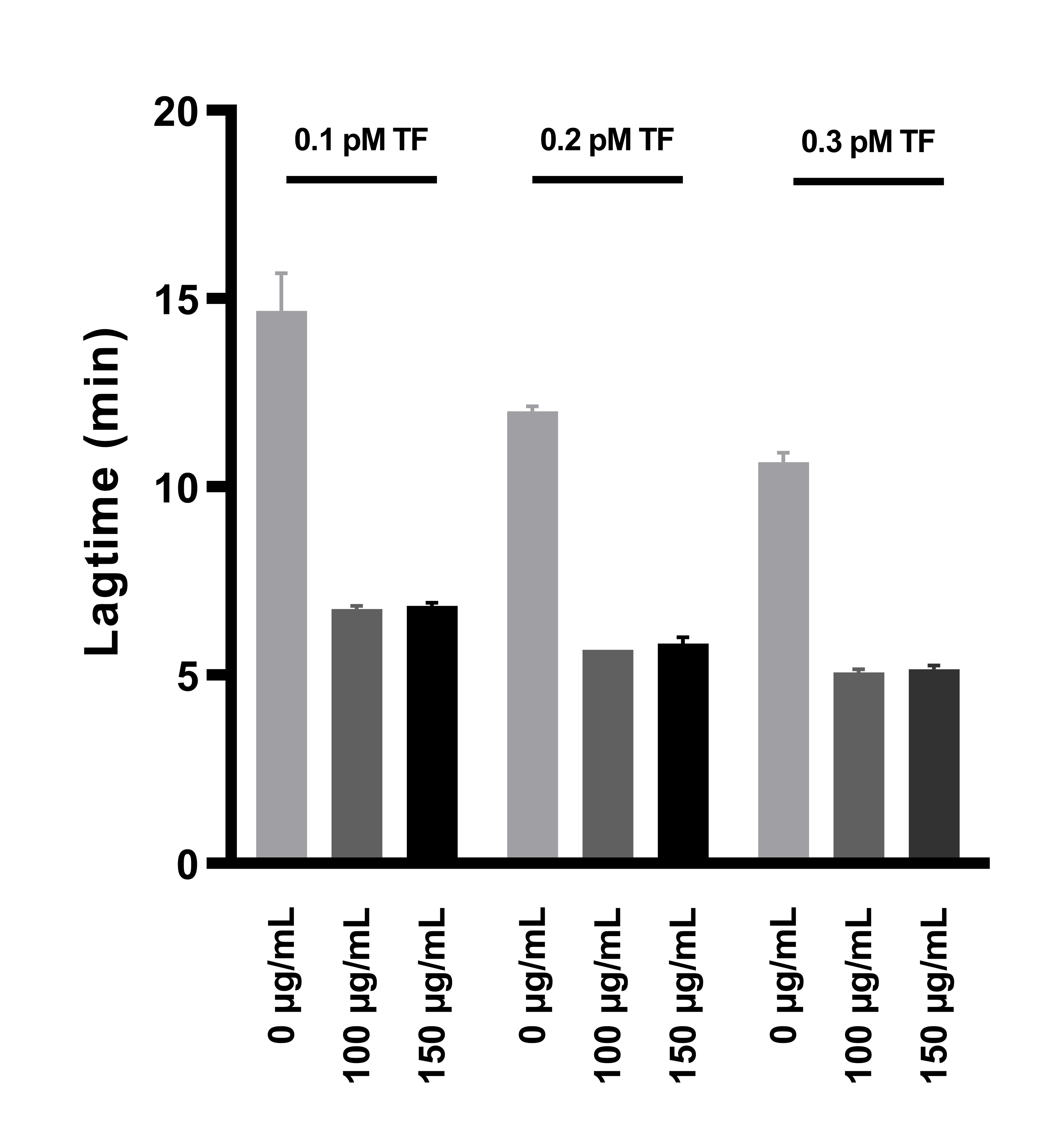

Supplement: S4 Fig — Addition of 100 μg/L or 150 μg/L aTFPI reduce LT in samples with 0.1, 0.2 or 0.3 pM TF considerably but no difference between 100 and 150 μg/mL. The bars show mean and SD of 4 plasma samples. (TIF) [file pone.0288918.s004.tif]

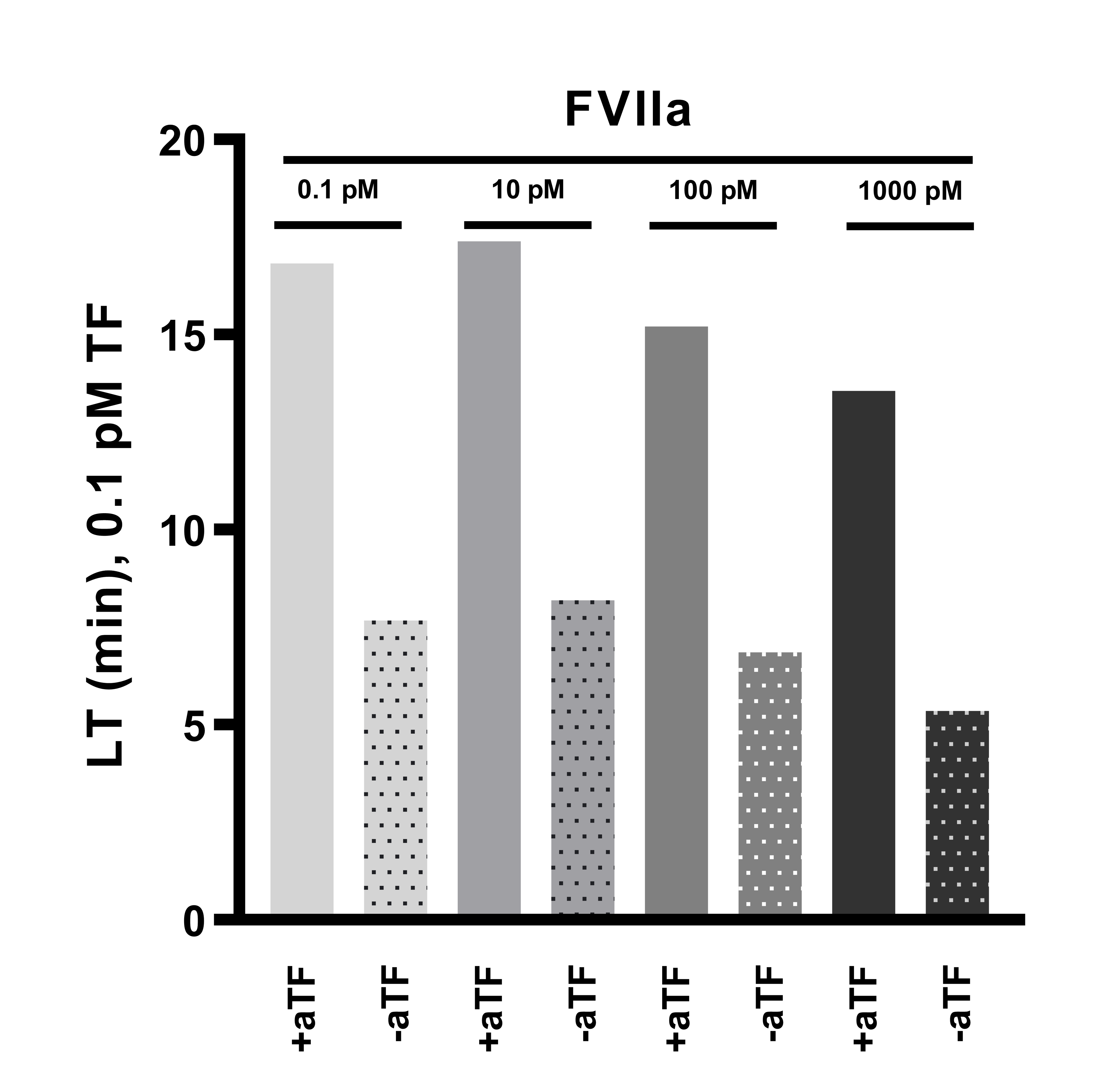

Supplement: S5 Fig — Addition of FVIIa reduces LT but it also reduces LT in the same sample in the presence of aTF, i.e. that the effect is not a TF dependent activity. (TIF) [file pone.0288918.s005.tif]

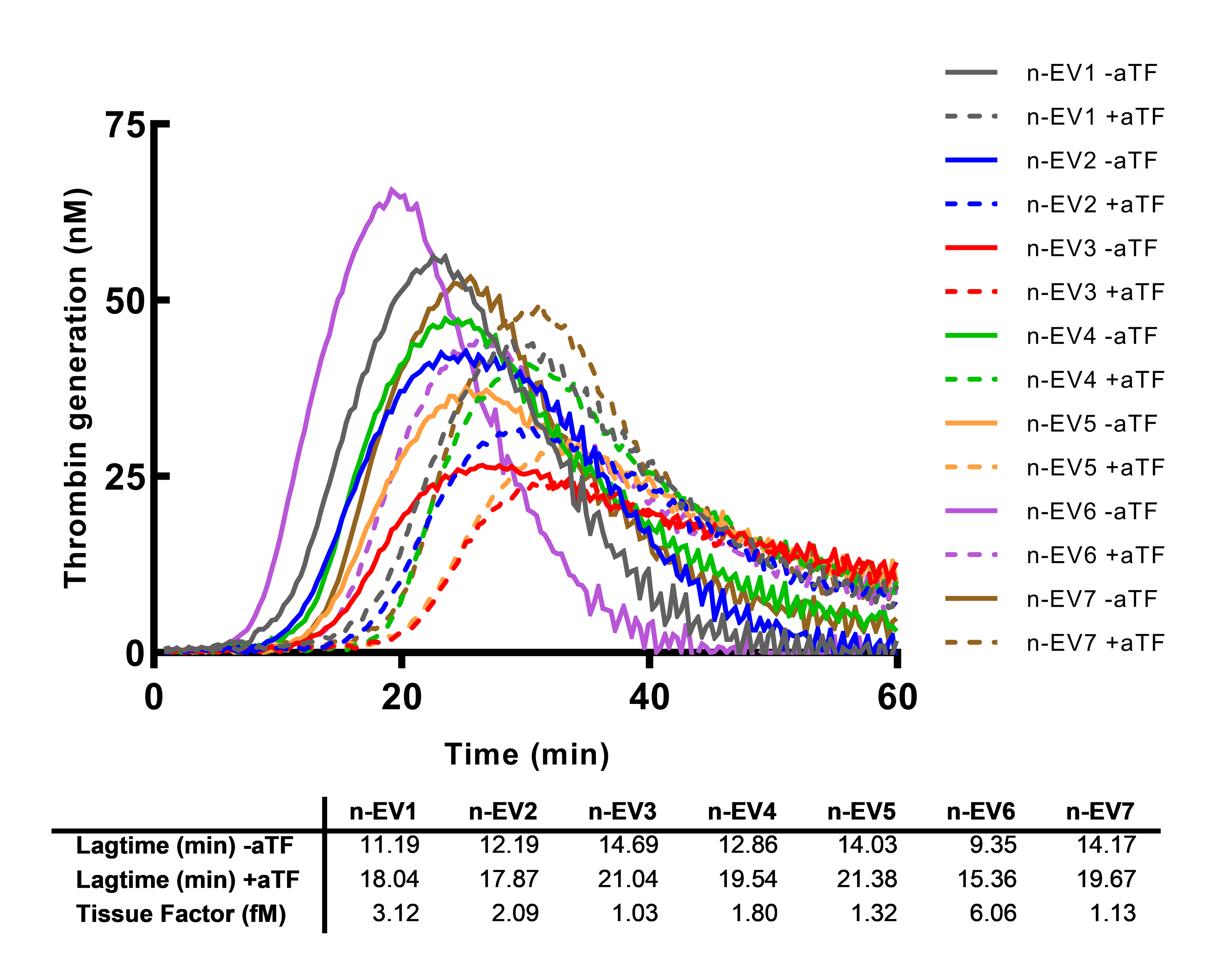

Supplement: S6 Fig — The same figure as Fig 4, but including all seven normal volunteers with TF activity of 1–6 pM. Below the figure is described LT in the presence and absence of aTF and the corresponding TF activity. (TIF) [file pone.0288918.s006.tif]

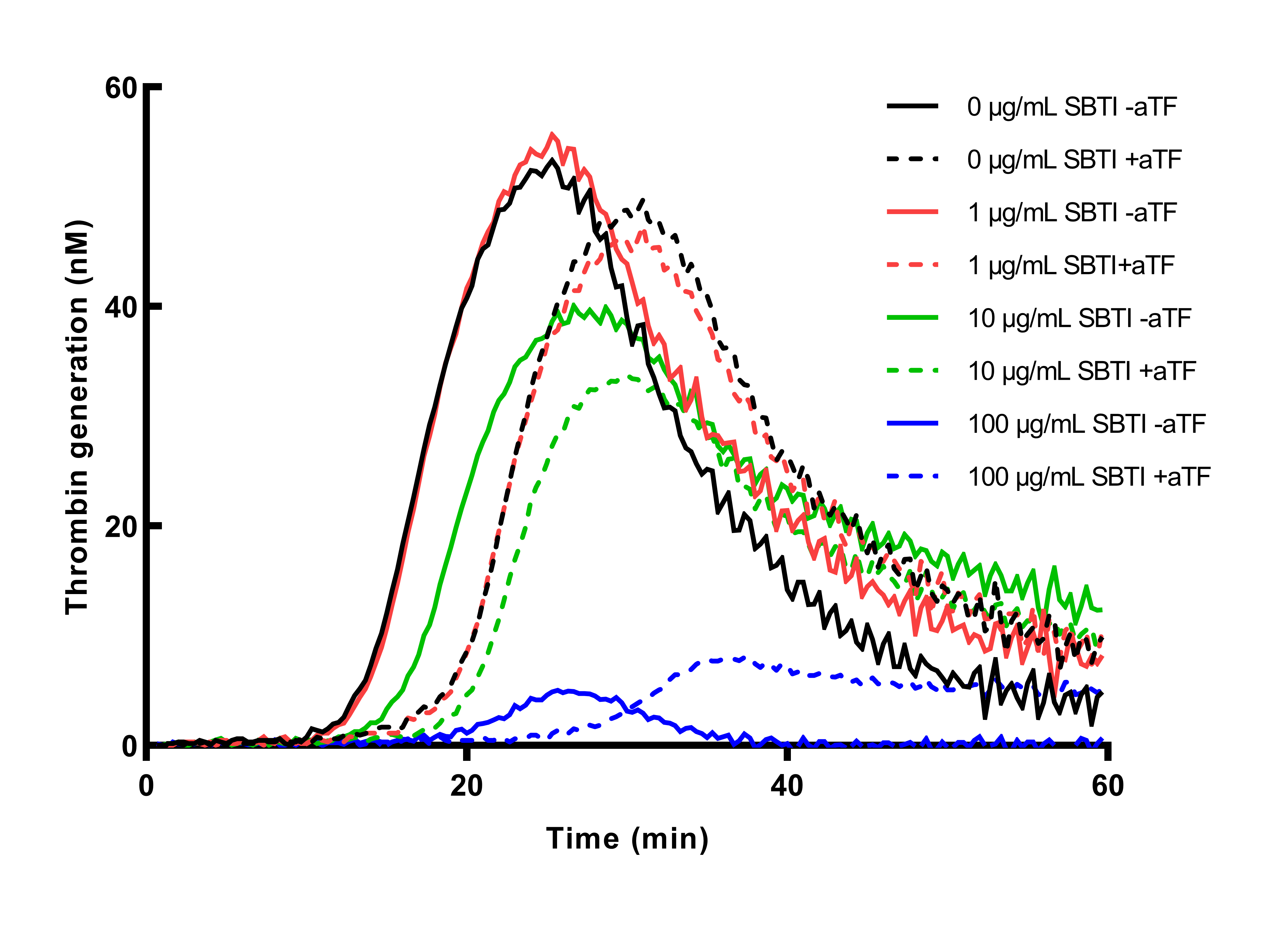

Supplement: S7 Fig — SBTI in various concentrations was added to a sample with 1 pM TF. It appears that the higher concentrations of SBTI inhibit the activity in samples without aTF as well as in the presence of aTF. (TIF) [file pone.0288918.s007.tif]
