# Supplementary material for: A sensitive tissue factor activity assay determined by an optimized thrombin generation method
Source: PLoS One. 2023 Jul 19;18(7):e0288918. doi: 10.1371/journal.pone.0288918 (PMC10355404; doi:10.1371/journal.pone.0288918)
Supplement: Attachment — Submitted filename: Response to reviewers.docx [file pone.0288918.s008.docx]

Response to reviewers

Journal Requirements:

When submitting your revision, we need you to address these additional requirements.

1. Please ensure that your manuscript meets PLOS ONE's style requirements, including those for file naming. The PLOS ONE style templates can be found at
   <https://journals.plos.org/plosone/s/file?id=wjVg/PLOSOne_formatting_sample_main_body.pdf> and 
   <https://journals.plos.org/plosone/s/file?id=ba62/PLOSOne_formatting_sample_title_authors_affiliations.pdf>

R: We have checked it, and we believe that the paper meets PLOS ONE’s requirements

2. Please provide additional details regarding participant consent. In the ethics statement in the Methods and online submission information, please ensure that you have specified what type you obtained (for instance, written or verbal, and if verbal, how it was documented and witnessed). If your study included minors, state whether you obtained consent from parents or guardians. If the need for consent was waived by the ethics committee, please include this information.

Additional Editor Comments:

The reviewers have spent a considerable amount of time providing careful review and commentary on this manuscript. They have submitted detailed feedback and recommendations which are aimed at improving the presentation of this work. If the authors address these comments, both reviewers are of the opinion that this paper would constitute a valuable contribution to scientific literature. Please address their comments by either improving the manuscript as suggested or by adding a justification for the original choices.

R: Thank you for the comments. We have made a thorough revision of the paper and commented each point below. We have complied with most of the points, but some of the statements in the criticism are actually nor correct, as you can see in our responses

Please note that the reviewers disagreed on whether the manuscript is presented in an intelligible fashion and written in standard English. Reviewer 1 provided detailed commentary on improving the organization of the paper and the presentation of the data - please address these comments if you choose to submit a revised version of the manuscript.

This is of course an important point to consider. We have made a substantial revision of the paper and relocated several parts of the paper. Furthermore, we have made a short description in the introduction of the points that have been investigated to establish the method, and in the Methods we have described these points in some detail. Consequently, we think (and hope) that it will be easier to follow the line of thoughts that we have followed when we developed the method. Thus, some of the suggestions have been followed but not all, as you can read in our responses to all the points raised. Although some of the points could be mentioned in Methods, it is actually part of the development of the method, and, therefore, also part of the Results. Consequently we think that it is more logical that the sequence of various points to consider is followed.

This editor and both reviewers are of the opinion that the statistical analysis requires improvement. Please consider requesting input from someone trained in statistical analysis of scientific data.

R: I have to respond to this request. I do not really agree. I am quite well trained and experienced using statistical analysis, but I agree that I do not use p-values very much (and in this respect I am in line with our professional statisticians). This is establishment of a method, not a test of a hypothesis. If you compare some groups, you have to use statistical analysis to decide whether they differ from each other or not. If you test some improvements of a method, you will usually see a clear difference. E.g., if you add antibodies against TFPI you will see a decrease of lagtime from 14 to 7 min reproducibly, and then you do not really need statistics (especially when you have a low variation of duplicates as it is in this assay) - I know that behind my assessments are the knowledge of e.g. Students t-test and when you have a large difference and a small variation, the t-value will be very high (i.e. a very low p-value). All changes and improvements have been tested carefully, and we have included CVs of the duplicates to describe the reproducibility of the test. With the inclusion of many results we think that all the decisions should be clear and unequivocal.

Reviewer 1 further makes the recommendation that the authors add a comparison to the Mackman lab in-house assay, published by Hisada et al. While this is sound advice and would strengthen this publication, it is not a requirement for resubmission of the manuscript.

I also want to make a comment to this. From first on I actually had the same idea, but we will probably not get exactly the same results, and which one is the correct one? We have used the Mackman-method in several studies, and although it certainly gives an estimation of the TF activity, I do not think that it is a perfect method. When we make that analysis, we measure the difference in absorption caused by the activated FXa, and this is done without and with addition of antibodies against TF. At low concentrations of TF activity we frequently got a higher level in the presence of these antibodies (it should of course be the other way round), and how could that happen? Perhaps it is caused by the unspecific effect of FXa as Østerud described in his recent paper. Therefore, I am not sure what we achieve from a comparison like that. Very recently, there was a comparison between the Mackman-method and the Østerud-method, and they got quite different results – it was performed in the Mackman-lab, and the results were questioned by the Østerud-lab (JTH 2023;21:1059-61 and 1063-64).

I can also tell that there has been a recent ISTH multicenter comparison of different methods to estimate TF activity. Several labs used the Mackman-method but there was quite a high variation between the labs. We participated with our method and it did quite well (this multicenter test will probably be published quite soon)

[Note: HTML markup is below. Please do not edit.]

Reviewers' comments:

Reviewer's Responses to Questions

**Comments to the Author**

1. Is the manuscript technically sound, and do the data support the conclusions?

The manuscript must describe a technically sound piece of scientific research with data that supports the conclusions. Experiments must have been conducted rigorously, with appropriate controls, replication, and sample sizes. The conclusions must be drawn appropriately based on the data presented.

Reviewer #1: Yes

Reviewer #2: Partly

2. Has the statistical analysis been performed appropriately and rigorously?

Reviewer #1: No

Reviewer #2: No

3. Have the authors made all data underlying the findings in their manuscript fully available?

The [PLOS Data policy](https://eur03.safelinks.protection.outlook.com/?url=http%3A%2F%2Fwww.plosone.org%2Fstatic%2Fpolicies.action%23sharing&data=05%7C01%7C%7Cb67714690d914c209ee508db16808714%7C5968b90c51a64f088b4750ffffbe2e4f%7C0%7C0%7C638128514783553815%7CUnknown%7CTWFpbGZsb3d8eyJWIjoiMC4wLjAwMDAiLCJQIjoiV2luMzIiLCJBTiI6Ik1haWwiLCJXVCI6Mn0%3D%7C3000%7C%7C%7C&sdata=cwz7E7nVOaoiTlUgPqJbMJdpgpEm3NTwVZwNQHmSXfQ%3D&reserved=0) requires authors to make all data underlying the findings described in their manuscript fully available without restriction, with rare exception (please refer to the Data Availability Statement in the manuscript PDF file). The data should be provided as part of the manuscript or its supporting information, or deposited to a public repository. For example, in addition to summary statistics, the data points behind means, medians and variance measures should be available. If there are restrictions on publicly sharing data—e.g. participant privacy or use of data from a third party—those must be specified.

Reviewer #1: No

Reviewer #2: No

4. Is the manuscript presented in an intelligible fashion and written in standard English?

PLOS ONE does not copyedit accepted manuscripts, so the language in submitted articles must be clear, correct, and unambiguous. Any typographical or grammatical errors should be corrected at revision, so please note any specific errors here.

Reviewer #1: No

Reviewer #2: Yes

5. Review Comments to the Author

Please use the space provided to explain your answers to the questions above. You may also include additional comments for the author, including concerns about dual publication, research ethics, or publication ethics. (Please upload your review as an attachment if it exceeds 20,000 characters)

Reviewer #1: The authors present a publication titled “A sensitive tissue factor activity assay determined by an optimized thrombin generation method”. The purpose of this publication as stated by the authors was to “establish a sensitive method to detect low levels of tissue factor (TF) based on the calibrated automated thrombogram (CAT) method”. This is important and relevant research to have simple measurement of the major initiator of the coagulation cascade. The study strengths are to use commercial grade reagents and a well-known method to rapidly determine tissue factor activity in plasma samples. This test would hopefully be an important step to bring measure of tissue factor from the research bench using chromatography assays to more of a commercial test. Despite these strengths, the paper is poorly written, organized, and fails to provide the data to be sufficient for publication in current form. Please see general comments and specific comments below:

The first main weakness in this paper is poor presentation to support their findings. Although the authors present graphical examples of the seven donors tested they fail to supply table listing the demographics of these donors (all men? how old?). Another table in the paper or supplemental data should list the computed CAT values and the CVs of each of the runs. This prevents a detailed review of the papers finding which is hard to get from the graphs. Without this date it is difficult to determine the CVs independently. Also help in understanding if additional donor testing is needed to reduce the CV to less than 10%, which is needed for high-quality research assay. Therefore, it is recommending the authors present this data and consider increasing the number of donors to reduce the CV value.

R: We have now included several supplementary figures to elucidate how the method was established. Increasing the number of donors will not lower the CV, which is the variation of each of the determinations.

As there are great number of tests out their increasing the number of donors documenting the sensitivity and specificity of the test is important. This is needed despite previously published low donor tested Hellum et al (only three donors) and Osterund et al (did not report how many). Furthermore, sex and age and other pertinent demographic variables are important for rigor and reproducibility in research. Although, Scully et al (J Thromb Thrombolysis, 2009) documented no difference in monocyte expression of TF between men and women. The NIH states that we should always consider sex, race, and other characteristics as biological variable. . Lastly, the authors should report sensitivity and specificity in predicting a TF expression in a donor using their test, which is necessary to compare tests.

Furthermore, as many studies have been published to measure tissue factor activity, it would be good for the authors to review the publications, cite from Mackman group, and discuss other methods in their paper including:

1. Mackman N, Sachetto ATA, Hisada Y. Measurement of tissue factor-positive extracellular vesicles in plasma: strengths and weaknesses of current methods. Curr Opin Hematol. 2022 Sep 1;29(5):266-274. doi: 10.1097/MOH.0000000000000730. Epub 2022 Jul 11. PMID: 35852819.
2. Hisada Y, Mackman N. Measurement of tissue factor activity in extracellular vesicles from human plasma samples. Res Pract Thromb Haemost. 2018 Nov 20;3(1):44-48. doi: 10.1002/rth2.12165. PMID: 30656275; PMCID: PMC6332748.

The first is excellent review that should be cite and evaluated in reporting a novel sensitive test. The second paper (Hisada et al) is the publication of the Mackman lab in-house assay which would strengthen this publication if the authors were to add a comparison to this test. This reviewer recommends repeating the study to compare these methods. Otherwise, this paper will not have the impact it needs to move toward a commercial test.

I know the papers. Since we wanted to focus on our method I did not refer to several other methods, but I have now included more text on this and used the Mackman paper as a reference. The second paper actually describes the same as the paper we have referenced (#7 in the first version), and the paper by Vallier (#6) describes an improved version of the same method. But we have now included this paper as well. We do not think that it would be appropriate to include several other papers to discuss measurement of TF activity – the scope of this paper was to describe a rather simple method to measure TF activity, not to review the whole field of methodologies.

Regarding a comparison: please see the initial comments.

Lastly, explain why an increased phospholipids would increase the lag time. As Mackman et al states above phospholipids that come from cellular activation (especially phosphatidylserine) activate cryptic TF to become active TF. This would lead to a decrease in lag time. Please explain why you found an increase in lagtime or was it not significant because of high CV.

R: We certainly do not find an increased lagtime when we add PPL. It decreases as you write, and that is what was written in the paper (fig 5 and text in Results)

Overall, figure legends should be at the end or connected to figures not in the text. All acronyms need to be spelled out. Overall, methods are very disorganized and large sections are missing compared to recent Osterund et al paper included NTA analysis and other details on the research process.

R: Figure legends are inserted into the text as recommended by the Journal: “ Figure captions are inserted immediately after the first paragraph in which the figure is cited. Figure files are uploaded separately.”

This method relies on known methodology, and, therefore, it has been described shortly. And I agree that it was too short. We have now expanded the text and especially included a section to describe the line in developing the methodology. We have not included NTA analysis in this paper, as it has not been described in most other papers measuring TF (e.g. the Mackman papers, Vallier et al). We have previously used the same centrifugation steps and described the results with western blots, NTA and electronmicroscopy in several papers.

ABSTRACT:
Line 22: Tissue factor concentration is different than activity as you are measuring only activity it's important to mention that. You are not measuring the total amount of tissue factor, the active vs. non-active (aka cryptic).

R: Yes we totally agree. But this first sentence means generally (including activity as well as mass concentration). The next sentence describes what we want measure, and we have stressed that TF activity is the relevant measurement.
Line 23: Are you determining the sensitivity and specificity of your assay or are you studying the CV?

R: The words sensitivity and specificity can be used in different connections. Sensitivity probably means the ability to measure low concentrations. In the paper is written “sensitive” because we can reliably measure very low concentrations. Specificity means that you measure specifically TF activity and not some other activity. This is ensured using antibodies against TF – we get a signal from the sample and this is almost completely inhibited using aTF. So, when we want to establish a method this is what we show and have shown in the paper (this is implicit in establishing a method). The variation is another part of describing the method.

Line 25: Please correc that the assay is based on “Thrombin generation”, it's based on :”lag time” to thrombin generation

R: We do not agree. The method is based on TG (which is an assay) of which we use the lagtime for estimations which is written two lines below.
Line 28: define lag time

R: We cannot make all descriptions in an abstract. Lagtime is very well known from readers who have used TG.
Line 30: define calibrators

R: “Calibrators” is a very well-known word and well-defined

Line 31: This should be in methods: “Contact activation of the coagulation system was avoided using CTI plasma samples in Monovette tubes.”

R: This is part of the development of the method and we think that it is an important part of the results. Otherwise you could actually put all the results into the method section, since it is development of a reliable method.

Line 34: There is no mention on the methods of how you measured EV concentrations. Clarify EV up concentrated as this is not a standard term

R: As is usually the case measuring TF, we have not measured EV concentrations (we have done this in many other papers). “Concentrated” means that the EVs are solubilized in a smaller volume than the original volume of plasma from which they were centrifuged (this is now explained in the text)

Line 35: “However, the step with isolation of EVs have a higher inherent CV.” This is not appropriate for abstract but should be in the limitations.

R: I do not agree. I think it is an important sentence in the abstract
Line 37: Precise would be all CV less than 10% for research-based assay and less than 5% for diagnostic assays. I would remove this word.

R: Measuring EV-TF has a high variation, so in comparison with other methods it was rather precise.

INTRODUCTION:
Line 42: Tissue factor is the PRIMARY coagulation factor that activates the coagulation system, please see papers on cell-based coagulation

R:. We agree and have expanded the text.

Line 43: Do you mean increased expression of tissue factor?

R: Cancer cells express the gene for TF which leads to an increased activity in plasma as described in the paper. It is the presence of TF in plasma which has been suggested to increase the risk of thrombosis

Line 45: What do you mean present inside vascular walls, do you mean endothelial cell?

R: No. TF is expressed in cells below endothelial cells, usually called perivascular cells. Endothelial cells do not synthesize TF in the normal state, but they may be activated to express TF (it is controversial whether it happens in vivo).

Line 48: What do you mean the measurement of TF, protein, or activity?

R: As it is clear from the text you could potentially make different measurements, but this is explained in the next lines.

Line 61: Please discuss the Yohei paper as referenced above

R: it has been referenced in the introduction now – and earlier in Introduction the text has been expanded

Line 62: Please clarify the purpose of the hypothesis is it to determine the sensitivity of the test or accuracy (CV) and is it for protein expression or tissue factor activity

R: The purpose of the hypothesis? I do not understand? The aim is as described: to establish a method measuring TF activity using TG. This includes optimization of various additions and description of linearity, sensitivity and variation. (Accuracy is not the same as CV which is variation. Accuracy is the trueness of the measurement (is it the correct value that it measures – this can only be estimated if you have a reference material or reference method))

MATERIALS AND METHODS:
Line 67: Please make materials section into one paragraph

R: OK, this has been done
Line 81: Most TF activity assays discard the first three ml of blood draw, why was this not followed?

R. It was certainly done, as we always do when we do measurements in the coagulation lab. We apologize for having not written this
Line 95: Why did you do CAT measurements in duplicates? The thrombinoscope software recommends triplicates.

R: It is correct that triplicates have been recommended, but this does not give much sense for a clinical biochemist. We use duplicates, and using triplicates do not reduce the variation much (CV is reduced to 81 %) but increases the expense 50 %. We actually have a small variation on the duplicates, and triplicates would make a negligible improvement. CVs in the established method has been inserted in the text in Results
Line 99: Spell out all acronyms…ETP

R: OK we have done that. We apologize for this fault
Line 104: Please state the standard dilution of the MP reagent, not spelled out acronym

R: The reagent is called MP reagent if you will purchase it

Line 107: Please clarify that the incubation with aTF was done before CAT or is it wrong section of the methods

R: It has now been stated that it is before analysis

Line 113: Why no sensitivity or specificity calculation as you did a test on TF EV from donor blood.

R: I do not know what the reviewer means. Sensitivity and specificity for what? It makes no sense

RESULT
Line 119: This is methods part and need to be moved up and explained.

R: We do not agree. This is also a result. All the work to establish an assay could be described in the Method section, but it is actually a result of all the work done.

Line 145: Watch out for acronyms like fig. please spell out

R: According to the recommendations from the Journal we have to write “fig”

Line 127-129: This explanation needs to go to the methods.

R: We agree that this could be written in Methods

Line 131 to 135 Please put into figure legend (put all figure descriptions into a legend)

R: This is a figure legend? It is put into the text as prescribed for the Journal?

Line 137 to 139: This is discussion and needs to be betterl explained

R: we describe the method in the order that we have tested various possibilities. This is a result but it is also discussed in Discussion, and we have discussed it in more detail

Line 149 to 151: Goes into methods

R: We agree that it is text to be written in methods (what it also was). However, we think it makes sense to repeat it here (shortly) in connection with the results. Otherwise the readers will probably not understand the text

Line 151: How were EVs concentration measured, this needs to be put in methods

R: EVs were not counted

Line 153: Were not all samples treated the same? Why are higher ones achieved with LPS?

R: I am not sure what this comment means? Incubation with LPS stimulates monocytes which release EV-TF, and therefore TF activity is higher in LPS-stimulated samples. Using LPS-stimulated samples is a standard procedure when you want to create samples with a high TF activity

Line 153: we need a table describing the age, gender, and others to help repeat study

R: We have now included a sentence describing age and gender of the group. Because of the low number, we cannot describe it more detailed according to Danish law regarding personal data security

Line 165: Phospholipids usually activate TF, please explain in discussion why would lag time be prolonged with increased phospholipids

R: I do not understand this mistake, PPL increase TG, and therefore you may get a shorter lagtime. Lagtime is not prolonged as also written in the text.

Line 175: a table needed to list all of this data, the CV and other can be in supplemental.

R: We admit that we have not done an extensive investigation of variation. But from the results we can give an estimate which is usable for researchers who want to use the method. We do think that it is easier to describe and explain the results in the way it has been done. The estimates of CVs are important to know for researchers and it is a normal procedure when you describe a method.

Line 179-181: As the variation is high it would be good to test vs. a published assay (Hisada et al) for reference.

R: Please see our initial comments regarding this comparison. The variation is not high compared with other published papers measuring TF activity

For graphs and figures be sure your legends are present and spelled out in figures.

DISCUSSION:
Line 195: Can you comment on the CTI differences between 18.3 and 36.6 ug/mL as the data is not presented. Can it be a difference because the first 3 ML of blood was not discarded as performed in other studies.

R: We have included the results for this comparison. As mentioned before the first tube has been discarded

Line 198-201: Very long sentence, please revise in two or three sentences.

R: We agree. We have separated in two sentences
Line 201-208: This is a list of papers with no relation of their findings with the results

R: This is a list of previous publications using TG to measure TF activity. You usually must include previous research on the same topic, also stressing that although they used the technique, they did not use it to make a precise TF activity as in this publication. It underlines that we are aware of previous publications from which we have based this methodology and that we have developed a method not described before

Line 208-211: Rephrase to say we chose the previous method to include in our study as..

R: I do not understand this comment. I think that the text describes what we have done

Line 221: Evs are made up of phospholipids. How do you distinguish that one of the donors EVS do not contribute the phospholipids to override the system. How many of the EVs were leukocytes vs. platelets?Platelet EVs do not contain TF but are more numerous that leukocyte

R: That is the reason why we made the test of addition of PPL. We know that there is a large difference when we use reagents without PPL compared with reagents containing PPL, and, consequently, the amount of PPL from EVs may have a role. However, we use reagents with 4 µmol/L PPL in order to measure TF activity. If we increase the concentration of PPL to 8 mmol/L we only see a small effect at the lowest TF concentrations (figure 5). Since this is a higher amount of PPL than expected from EVs, the PPL from EVs will only have a negligible effect.

No, we have not investigated the origin of the EVs – this was not the purpose. We only wanted to measure TF activity in plasma from normal healthy persons.

Line 224-225: Should this be iits own paragraph?

R: We have now expanded the paragraph

Line 230: Did you do test with soybean trypsin inhibitor? Nothing was reported in results. please include the data in supplemental.

R: Yes thank you for this comment. The results have now been included
Line 236-237: Please see line 224-225

R: We have merged with the following paragraph

Line 238: Please report your CVs for all values and then mention the ones for Vallier so we can know the actual difference. If your CVs are not less than 10% for lower TF, then how can you call this improved?

R: It is certainly not easy to describe the results from Vallier in a table – it is described in a table, but you cannot really see the levels that were measured. If we measure TF activity between 0.1 and 0.8 pM we had a CV below 10 %. At the very low concentration in normal plasma, it was considerable higher, but very few methods can detect this low level so you can certainly not expect that it should be possible to get a CV below 10 %. That would be very unlikely – no one has ever done that.

Line 243: Is it possible they found no TF in normal controls because they discarded the first 3 mL?

R: No, we did that as well
Line 246: This conclusion is too short and does not have a limitation section.

R: You usually do not include limitations in the conclusion. The limitations have been described in the text before the conclusion. A conclusion is normally short

Reviewer #2: Summary: The authors present an interesting study detailing a novel method for determination of tissue factor (TF) concentration utilizing a commercially available thrombin generation assay (CAT; STAGO). Development of such an assay would make quantification of TF more readily available for both clinical and research investigations.

R: Thank you for this remark
While the overarching objective of the study, to measure TF based on TG, is clear, the specific sub-aims/experimental procedures implemented are not clearly defined in the introduction or methods sections. There is no study hypothesis. Currently, the manuscript is missing a clear delineation of Methods/Results/Discussion as portions of each of these sections are presented in different areas/not clearly in a sequential order. The reader is left to go back and forth between sections for clarity at several points. It would be useful to have 1-2 sentences in the introduction clarifying how the authors planned to go about developing and evaluating the TG based TF detection method, and what conditions/variables will be assessed in this investigation – as this is not readily apparent.

R: Thank you for this comment. We agree that the text has been too short. We have now described the procedure of tests in the introduction (shortly) and in more detail in the Methods so that the sequence of results hopefully is more logical and understandable.

Further, the statistical methods are lacking. The sample sizes are not clearly reported, and only representative data displayed. It is not stated whether a power analysis was performed or whether the study was accurately powered to assess each of the outcome measures investigated. At various points throughout the manuscript the authors mentioned that they evaluated an experimental condition – but there is no data shown to support this. The manuscript could strongly benefit from a detailed Supplemental Methods and Supplemental Results document so that all data and experimental procedures referenced are accounted for and presented. It would be important to perform a comparative study to assess TF using the TG method developed in this study alongside other reference methods, prior to making claims that this is an “established” method.

R: Thank you also for this comment. We have now included several results as supplementary data so that it is easy to follow the conclusions. We agree that we have not used statistical methods (as also described in our initial comments). However, establishing a method is to examine which conditions are of importance and which are not. There may be a small difference which could be significant if you make a high number of tests, but that is not very interesting – very small effects are probably not important for the method. E.g. if you examine the effect of aTFPI (now included in supplemental), you get a substantial effect, many times higher than the variation. You could of course make a t-test or some other test which would be highly significant, but in our opinion, it is evident from the results. But we have now included several supplemental data and included a description of variation in the method after development

We agree that “established method” is perhaps ambitious, but in our opinion the present method can be used reasonably to quantitate TF activity. Unfortunately, there is no “reference method” and no “reference material” to measure the level of TF. The method from Mackman’s laboratory has been used by several researchers. However, Østerud et al made a powerful argumentation that this method may overestimate TF because FXa activates FXa, and therefore, a comparison will be difficult. We have actually used this method in previous projects, and there are some peculiarities in this technique – it can of course quantitate TF but is it the correct level? I am sure that you will see that low concentrations are low and high concentrations are high with both methods, but if there are some discrepancies between the methods which one is the correct one? I can tell that we have recently participated in a multicenter testing of various TF assays, and the variation between methods is large, also if you use the same kind of test. Please, also see our initial comments.

We have commented on the arguments below on each of them.

Additional comments:

Methods:

How many healthy donors were included in the donor pool for preparation of the SP? Likely the experiment should be repeated additional times with SP prepared from separate donor pools. Likewise, may be important to repeat the study using SP prepared from patients with pathologies subject to TF investigation.

R: We have done the experiments several times (actually very many times, because of various modifications during the period). We have included 5-10 normal, healthy persons in the pools that we have used – a pool was produced for each series of investigations. We always include calibrators (i.e. dilutions of PPP Low reagent), and the differences in lagtime are actually not much different using different SPs. But if we should perform the assay for a particular project we would produce a large amount in order to reduce a potential difference between SPs. This has now been described in the Discussion. I do not think that we should use pathologic samples since the purpose is a stable source of other coagulation factors than TF

The method section could benefit from a table that indicates what additives /components were utilized in the sample mixtures for each of the experiments performed (ex. aTFPI, SP, aTF, EV suspension, CTI, etc.)

R: We have now explicitly described quite thoroughly the series of experiments and the concentrations used, so we hope (and think) that it is more obvious. We got a basic set-up which was developed and optimized during all the initial tests of different additions which are written in more detail. We think that it will be difficult to make a reasonable table, e.g. after testing the effect of aTFPI we included that in all the remaining investigations etc.

How was the concentration of CTI to be used decided upon? *Update - this is mentioned later in discussion but should be clarified in the methods earlier and cited appropriately. Were other concentrations tested by these authors? And where are those results reported?

R: We agree that it could be confusing. As just mentioned we have thoroughly described the series of experiments and also mentioned the concentrations of various additions, which subsequently are discussed in the Discussion section.

Throughout methods and results it is difficult to ascertain how many times the experiments were repeated. The statistical analysis section in the methods states that representative data is presented, but throughout the manuscript the sample sizes are never clarified - it is only mentioned that "several" replicates were performed. The sample sizes should be clearly stated. Additionally, was a power analysis performed prior to the study? Or how were the sample sizes decided upon? Was the study accurately powered at each phase to draw the conclusions reported?

R: We have tried to clarify this further in the text. As described in the Method section all results in each test is performed in duplicates, and each substudy is performed several times at least 2-3 times but often more times. We are quite confident that you will agree on our decisions when you see all the data which now are included in Supplementary. In the figure legends to these supplementary figures it is written how many samples etc that were used. Furthermore, we have included the variation that we had of duplicates in the experiments

We do not use power calculations. That is appropriate if you want to compare some groups, cases and controls, in order to be certain that you can detect some difference. But when you for instance want to investigate the importance of adding aTFPI and you get a huge difference you cannot doubt that this is beneficial. If you had a smaller difference it could be necessary to repeat the investigation more times to be sure, and if the difference is negligible and you have seen that in 2 or 3 experiments it is of no use to repeat it many times – a small difference can be tested to be “significant” (p< 0.05) if you have many determinations but then it is not important for the assay. We look at trends and not at particular differences. E.g. if we add CTI we can see that all the samples get a longer lagtime (and no TG in the presence of aTF), and if we repeat the experiment we see the same – then it is absolutely certain that there is a difference (what is the chance that 10 or 20 results are changed in the same direction – it is very small). So all decisions are solid and reliable.

Results

Are there reference data or experimental data that could be added to a supplement to show the variability in LT when aTFPI was applied versus when it was not? How was the concentration of aTFPI decided on?

R: The concentration came from Hellum et al which now is written in the text. And we tested a higher concentration to make sure that we inhibited all TFPI (supplementary figure 4)

Results line 137-138 states, “Ten pM [FVIIa] had a small effect which increased at higher concentrations, but an effect was also seen in samples with aTF added….” – Where are these results? Could they be provided in a supplement? What did the authors define as “a small effect”/what was the outcome of interest here? LT? How many times was this experiment repeated?

R: We apologize for not having shown the results. They are now included as supplementary figure 5.

In Fig 2 and 3, again it is not clearly stated how many replicates were performed to generate the TF concentration vs LT calibration curve; or how many replicates were performed when assessing linearity of the dilutions of TF in the LPS stimulated samples.

R: In each experiment we include a calibration curve of which each point is determined as a duplicate. This has been done several times after the methodology was established and TF activity was measured in various samples, at least more than 10 times (this has been stated in the text). Linearity has been done fully in two different experiments, but several times dilutions have been measured showing consistency between the dilutions (which is the same as linearity).

In Figure 4, thrombin generation in EV samples from 3 normal controls having 1-3 fM TF is shown – but earlier in the results it states that levels of TF in the normal controls ranged from 1-6 fM. Why are results for 4-6 fM not shown? Why do the authors speculate that LT was prolonged with escalating TF concentration in Fig 4A? What were the actual TF concentrations for EV5-8 in Fig 4B?

R: We only included the three lowest values in order not to overload the figure – and because we wanted to show the low values which are hard to measure. We have now included the highest result also and made a supplemental figure with all the results (it is somewhat crowdy). We have also included LT for each determination and the corresponding concentration of TF activity. We do not understand the question regarding LT and TF activity. A higher TF concentration always resulted in a shorter LT. LT and TF concentration has now been described in the figure. Hopefully it will clarify all.

In Figure 5, the abbreviation “PPL” is used in the figure caption and manuscript, but the abbreviation “PL” is used on the figures themselves – this should be standardized.

R: We are sorry for this. We have changed the abbreviation to PPL (at an earlier stage we used the abbreviation PL)

Throughout the results, it may be useful to provide the numerical LTs – it is difficult to ascertain the LTs and variability in LT from the figures and CV alone, especially when no sample size is provided. The ETP/Peak are more clearly depicted in figure format; however, if LT is the sole outcome of interest it may be beneficial to report mean results in a table format with standard deviations and sample sizes. This information can be obtained in part from Figure 2A – however there are no error bars or samples sizes included in the figure to indicate how many replicates were performed to achieve a lagtime result at each TF concentration.

This has been included in the figures. It is described in the Methods that all measurements in a specific measurement was made in duplicate. At the end of Results we have described CVs of calibrators (duplicates of LT and also total variation). Furthermore, the total variation for TF measurements in plasma samples are also shown

Discussion

The discussion mentions that additional CTI concentrations were evaluated – where are these results/could the be provided in supplement?

It has now been included as a supplemental figure 3 – mentioned in Results and discussed in the Discussion

Lines 211-218 provide important information that should be explained in the methods section, rather than in discussion.

R: As explained in one of the first comments, we have now described more extensively, first what we want to investigate to optimize the method in the Íntroduction (shortly), and subsequently this is further explained in Methods including the various concentrations etc to help and clarify the strategy. The results are subsequently discussed. Line 211-18 actually sums up what we have done, what we have used from the references in the literature, and how that was used in the final method – but the series of experiments are now described in the Methods section. We hope that these extensions in Introduction and Methods will make it more understandable, and then we think that these lines in the discussion can remain - we find it logical to refer to all these details when discussing the literature on this subject.

Line 224-225 Where are these results shown?

R: As mentioned, they are now included as a supplementary figure 5

Line 230-233 Is this referring to the present study? If so, were these results reported?

R: Yes, you are right, they were not shown -the results have now been shown as a supplementary figure 7

Further discussion on how the presented novel method is potentially superior to currently available methods would be important. What are the next steps/future directions for this work?

R: We are actually confident that it is ready for use. It works with a reasonably low variation and is very reproducible. All the reagents are commercially available. We will use the method in some projects to come. We think that we have stated at the end of the paper before the conclusion that this method has some advantages compared to the previous methods. And at least it is easier and rather quick.

6. PLOS authors have the option to publish the peer review history of their article ([what does this mean?](https://eur03.safelinks.protection.outlook.com/?url=https%3A%2F%2Fjournals.plos.org%2Fplosone%2Fs%2Feditorial-and-peer-review-process%23loc-peer-review-history&data=05%7C01%7C%7Cb67714690d914c209ee508db16808714%7C5968b90c51a64f088b4750ffffbe2e4f%7C0%7C0%7C638128514783553815%7CUnknown%7CTWFpbGZsb3d8eyJWIjoiMC4wLjAwMDAiLCJQIjoiV2luMzIiLCJBTiI6Ik1haWwiLCJXVCI6Mn0%3D%7C3000%7C%7C%7C&sdata=lKQFP9GD%2FeXh8iODdlGBLUGL08wRzghFIEW8XUkpVmo%3D&reserved=0)). If published, this will include your full peer review and any attached files.

If you choose “no”, your identity will remain anonymous but your review may still be made public.

**Do you want your identity to be public for this peer review?** For information about this choice, including consent withdrawal, please see our [Privacy Policy](https://eur03.safelinks.protection.outlook.com/?url=https%3A%2F%2Fwww.plos.org%2Fprivacy-policy&data=05%7C01%7C%7Cb67714690d914c209ee508db16808714%7C5968b90c51a64f088b4750ffffbe2e4f%7C0%7C0%7C638128514783553815%7CUnknown%7CTWFpbGZsb3d8eyJWIjoiMC4wLjAwMDAiLCJQIjoiV2luMzIiLCJBTiI6Ik1haWwiLCJXVCI6Mn0%3D%7C3000%7C%7C%7C&sdata=rb8AthVDv0jxkTIkWZpjmR0fEEHB%2F9noOS9Nf26CsC8%3D&reserved=0).

Reviewer #1: **Yes:** Andrew D. Meyer, MD, MS

Reviewer #2: No

[NOTE: If reviewer comments were submitted as an attachment file, they will be attached to this email and accessible via the submission site. Please log into your account, locate the manuscript record, and check for the action link "View Attachments". If this link does not appear, there are no attachment files.]

While revising your submission, please upload your figure files to the Preflight Analysis and Conversion Engine (PACE) digital diagnostic tool, [https://pacev2.apexcovantage.com/](https://eur03.safelinks.protection.outlook.com/?url=https%3A%2F%2Fpacev2.apexcovantage.com%2F&data=05%7C01%7C%7Cb67714690d914c209ee508db16808714%7C5968b90c51a64f088b4750ffffbe2e4f%7C0%7C0%7C638128514783710054%7CUnknown%7CTWFpbGZsb3d8eyJWIjoiMC4wLjAwMDAiLCJQIjoiV2luMzIiLCJBTiI6Ik1haWwiLCJXVCI6Mn0%3D%7C3000%7C%7C%7C&sdata=CxrIpqbgcxi1%2FRuE1hdBY%2F7q%2FmfrcfVBVnlwU2KnCbg%3D&reserved=0). PACE helps ensure that figures meet PLOS requirements. To use PACE, you must first register as a user. Registration is free. Then, login and navigate to the UPLOAD tab, where you will find detailed instructions on how to use the tool. If you encounter any issues or have any questions when using PACE, please email PLOS at [figures@plos.org](mailto:figures@plos.org). Please note that Supporting Information files do not need this step.
