# Supplementary material for: A sensitive tissue factor activity assay determined by an optimized thrombin generation method
Source: PLoS One. 2023 Jul 19;18(7):e0288918. doi: 10.1371/journal.pone.0288918 (PMC10355404; doi:10.1371/journal.pone.0288918)
Supplement: Attachment — Submitted filename: Response to reviewers.docx [file pone.0288918.s009.docx]

Dear Dr. Kristensen,

Thank you for submitting your manuscript to PLOS ONE. After careful consideration, we feel that it has merit but does not fully meet PLOS ONE’s publication criteria as it currently stands. Therefore, we invite you to submit a revised version of the manuscript that addresses the points raised during the review process.

Please note that in response to your concerns about the reviewer comments regarding your original submission, we have requested the evaluation of a third reviewer whose paper you have cited in your submission. This reviewer (Reviewer #3) has provided comments regarding your work. Please address these comments in full prior to re-submitting your manuscript.

Please submit your revised manuscript by Aug 03 2023 11:59PM. If you will need more time than this to complete your revisions, please reply to this message or contact the journal office at [plosone@plos.org](mailto:plosone@plos.org). When you're ready to submit your revision, log on to <https://www.editorialmanager.com/pone/> and select the 'Submissions Needing Revision' folder to locate your manuscript file.

Please include the following items when submitting your revised manuscript:

- A rebuttal letter that responds to each point raised by the academic editor and reviewer(s). You should upload this letter as a separate file labeled 'Response to Reviewers'.
- A marked-up copy of your manuscript that highlights changes made to the original version. You should upload this as a separate file labeled 'Revised Manuscript with Track Changes'.
- An unmarked version of your revised paper without tracked changes. You should upload this as a separate file labeled 'Manuscript'.

If you would like to make changes to your financial disclosure, please include your updated statement in your cover letter. Guidelines for resubmitting your figure files are available below the reviewer comments at the end of this letter.

If applicable, we recommend that you deposit your laboratory protocols in protocols.io to enhance the reproducibility of your results. Protocols.io assigns your protocol its own identifier (DOI) so that it can be cited independently in the future. For instructions see: [https://journals.plos.org/plosone/s/submission-guidelines#loc-laboratory-protocols](https://eur03.safelinks.protection.outlook.com/?url=https%3A%2F%2Fjournals.plos.org%2Fplosone%2Fs%2Fsubmission-guidelines%23loc-laboratory-protocols&data=05%7C01%7C%7C4659163a30064e9c739008db71267b87%7C5968b90c51a64f088b4750ffffbe2e4f%7C0%7C0%7C638228182329558832%7CUnknown%7CTWFpbGZsb3d8eyJWIjoiMC4wLjAwMDAiLCJQIjoiV2luMzIiLCJBTiI6Ik1haWwiLCJXVCI6Mn0%3D%7C3000%7C%7C%7C&sdata=v8FkxHCaQIaRbsNO6bxvavMr79gC%2FTW2KeYpp7uY9%2BI%3D&reserved=0). Additionally, PLOS ONE offers an option for publishing peer-reviewed Lab Protocol articles, which describe protocols hosted on protocols.io. Read more information on sharing protocols at [https://plos.org/protocols?utm_medium=editorial-email&utm_source=authorletters&utm_campaign=protocols](https://eur03.safelinks.protection.outlook.com/?url=https%3A%2F%2Fplos.org%2Fprotocols%3Futm_medium%3Deditorial-email%26utm_source%3Dauthorletters%26utm_campaign%3Dprotocols&data=05%7C01%7C%7C4659163a30064e9c739008db71267b87%7C5968b90c51a64f088b4750ffffbe2e4f%7C0%7C0%7C638228182329558832%7CUnknown%7CTWFpbGZsb3d8eyJWIjoiMC4wLjAwMDAiLCJQIjoiV2luMzIiLCJBTiI6Ik1haWwiLCJXVCI6Mn0%3D%7C3000%7C%7C%7C&sdata=ue4f9SQaT6DVmNcP8eRmofAzpdTwWIYS2ocOiAXnLAs%3D&reserved=0).

We look forward to receiving your revised manuscript.

Kind regards,

Heather Faith Pidcoke, MD, MSCI, PhD
Academic Editor
PLOS ONE

Journal Requirements:

Please review your reference list to ensure that it is complete and correct. If you have cited papers that have been retracted, please include the rationale for doing so in the manuscript text, or remove these references and replace them with relevant current references. Any changes to the reference list should be mentioned in the rebuttal letter that accompanies your revised manuscript. If you need to cite a retracted article, indicate the article’s retracted status in the References list and also include a citation and full reference for the retraction notice.


[Note: HTML markup is below. Please do not edit.]

Reviewers' comments:

Reviewer's Responses to Questions

**Comments to the Author**

1. If the authors have adequately addressed your comments raised in a previous round of review and you feel that this manuscript is now acceptable for publication, you may indicate that here to bypass the “Comments to the Author” section, enter your conflict of interest statement in the “Confidential to Editor” section, and submit your "Accept" recommendation.

Reviewer #1: All comments have been addressed

Reviewer #3: (No Response)

2. Is the manuscript technically sound, and do the data support the conclusions?

The manuscript must describe a technically sound piece of scientific research with data that supports the conclusions. Experiments must have been conducted rigorously, with appropriate controls, replication, and sample sizes. The conclusions must be drawn appropriately based on the data presented.

Reviewer #1: Yes

Reviewer #3: Partly

3. Has the statistical analysis been performed appropriately and rigorously?

Reviewer #1: Yes

Reviewer #3: I Don't Know

4. Have the authors made all data underlying the findings in their manuscript fully available?

The [PLOS Data policy](https://eur03.safelinks.protection.outlook.com/?url=http%3A%2F%2Fwww.plosone.org%2Fstatic%2Fpolicies.action%23sharing&data=05%7C01%7C%7C4659163a30064e9c739008db71267b87%7C5968b90c51a64f088b4750ffffbe2e4f%7C0%7C0%7C638228182329558832%7CUnknown%7CTWFpbGZsb3d8eyJWIjoiMC4wLjAwMDAiLCJQIjoiV2luMzIiLCJBTiI6Ik1haWwiLCJXVCI6Mn0%3D%7C3000%7C%7C%7C&sdata=ZjOB%2FrM4rLD%2FLMrUAfMjpTo1oFFBY%2F8RhP7cxQU6g3k%3D&reserved=0) requires authors to make all data underlying the findings described in their manuscript fully available without restriction, with rare exception (please refer to the Data Availability Statement in the manuscript PDF file). The data should be provided as part of the manuscript or its supporting information, or deposited to a public repository. For example, in addition to summary statistics, the data points behind means, medians and variance measures should be available. If there are restrictions on publicly sharing data—e.g. participant privacy or use of data from a third party—those must be specified.

Reviewer #1: Yes

Reviewer #3: Yes

5. Is the manuscript presented in an intelligible fashion and written in standard English?

PLOS ONE does not copyedit accepted manuscripts, so the language in submitted articles must be clear, correct, and unambiguous. Any typographical or grammatical errors should be corrected at revision, so please note any specific errors here.

Reviewer #1: Yes

Reviewer #3: Yes

6. Review Comments to the Author

Please use the space provided to explain your answers to the questions above. You may also include additional comments for the author, including concerns about dual publication, research ethics, or publication ethics. (Please upload your review as an attachment if it exceeds 20,000 characters)

Reviewer #1: (No Response)

Reviewer #3: It would have been great to have a reliable sensitive and specific assay for the measurement of TF activity in blood/plasma. However, the present assay is not any easier and cheaper assay for TF activity than the present assays available today. It is rather complicated as the EVs must be isolated, and then they are added back to a pool of plasma from different individuals. To avoid any plasma contribution to thrombin generation, they are adding CTI to block the intrinsic pathway and aTFPI to avoid plasma inhibition of TF activity, both expensive reagents.

R: It is correct that it is not very simple or cheap – and this has certainly not been stated in the paper. EVs must be isolated as in other assays for TF activity, but the advantage is that you can use thrombin generation (or CAT) which many researchers have admittance to do. Unlike the two most used methods (as described in the Introduction) you do not have to buy several different reagents which is not always easy and uncomplicated (which I have learned from a long life in the lab). In this respect it is more straightforward to use the CAT technique with few modifications, and in this way it is an alternative to other methods. An optimized method for TF activity measurement based on CAT has not been described before.

In the abstract, line 37, the authors state: “addition of PPL only increased lagtime slightly at very low concentrations of TF showing--” How can this be as they in the section 231-237, and Fig 5, shows that TG is enhanced with adding larger amounts of PPL, and LT is slightly shorter at a concentration of 10 fM TF. This does not make sense and should be corrected.

R: We have now explicitly described the effect on the estimation of TF activity concentration

It looks like detection of TF activity in plasma of healthy individuals, is the major goal of this study. To detect the TF activity, they concentrate the EVs up to 25 times, compared to the level of EVs in the plasma. This gives rise to very high levels of PPL that will cause more non-specific TF activity.

No, it is not very important to measure TF activity in normal, healthy individuals. As stated in the introduction it may be interesting to quantitate TF activity in cancer patients and other procoagulant patients. We have done this in some cancer patients previously and using the Mackman method it is not possible to detect an activity in all the patients. It could, therefore, be advantageously to measure lower concentrations. In their optimized method (Ref. #10 in the paper) they could actually measure TF activity in some of the healthy controls, and we therefore found it interesting that it was also possible with this method if we concentrated the EVs. But we believe that the main application of a method like this will be for patients who have a higher TF concentration. EVs were not concentrated 25 times because in the assay 20 µL of this solution is added to 60 µL SP, as described in Methods – we have now added this in the text. Thus it is concentrated about 6 times compared to normal plasma, and in the reaction it is further diluted to two thirds.

As we have described in the paper, high levels of PPL and FVIIa can activate FX, but this is the reason why we have tested the effect of increasing PPL in the assay which have a minor effect. What is “very high levels of PPL”? I do not think that we have very clear estimations of PPL concentrations of EVs. According to measurements with Zymuphene MP activity assay it should be at or below 10 nM PS equivalents. So even if the EVs are concentrated and only part of PPL in the reagent has PS activity, the amount of PPL from EVs cannot be more than the 4µM that we added.

But what else can be associated with the large amounts of EVs? There are several other procoagulant activities associated with EVs as for example FVIIa, FVa, FIXa and FXIa, that may contribute to shorter LT, especially at low concentrations of TF.

As stated, there is not a large amount of EVs in the assay, especially not when we measure higher concentrations of TF. There is no reference to these statements? We are not aware of papers that have estimated the concentration of these factors. It must be remembered that EVs are added to a standard plasma with normal concentrations of coagulation factors so this addition will probably be negligible. Even if it is correct, it will only be a problem at very low TF activities. Otherwise, it will also be a problem in other methods where EVs are added. Perhaps the reviewer means that the EVs have a procoagulant surface? That is exactly the reason why we tested the effect of adding phospholipids (fig 5). And as shown by this experiment, this is only a minor problem at low TF concentrations.

I do not believe that subtracting values obtained with aTF from results without aTF, is giving the right answer for the presence of TF activity or not.

This may be true, but it is a way to try to correct for it. We have now explicitly described the premise for this procedure, and also a possible uncertainty of the estimations because of this. In all the methods for TF activity there is some “blank value” which must be corrected for, and this way of correction may not be true in any of the methods. In any case, it only seems to be a potential interference at a very low TF activity as discussed explicitly in the paper now.

As the authors themselves states in lines 235-237: “Therefore, when measuring TF using EVs with procoagulant phospholipids, a potential increase of PPL concentrations has a very small effect of the measurement, but very low concentrations of TF may be overestimated”. I agree and believe that the detected TF activity in this assay system in EVs of healthy individuals, has nothing to do with TF activity. To disprove this, they must rule out the contribution of other procoagulant activities associated with highly concentrated EVs in the generation of thrombin.

Firstly, it is only when measuring TF activity in normal healthy persons that EVs are concentrated, and it is more relevant to measure higher concentrations in procoagulant persons. Secondly, we have participated in a trial of measurements of TF activity and the present method performed quite well. Making a measurement without and with aTF is usually what has been done to measure TF activity. Furthermore, I can tell that we have used the Mackman-method previously in samples from cancer patients – here we determine an activity with and without aTF. Frequently we got a higher activity with aTF included, i.e. we actually found a “negative concentration”. The present method seems to be much more reliable for lower concentrations.

I think it is a revealing statement that the measured TF activity has nothing to do with TF activity. We measure an activity which is inhibited by aTF. It may be slightly inaccurate but certainly not completely misleading – this is nonsense. How can aTF inhibit an activation which is not performed by TF?

FVIIa has different ways to mediate thrombin generation in plasma, and to assure that FVIIa is not involved in the generation of thrombin in the present assay in the absence of TF, they should use FVIIai to block the extrinsic pathway.

As shown in fig 1B there is no thrombin generation in the absence of TF. We have actually tried to add FVIIa and concentrations up to 10 pM did not result in a shorter lagtime, and 100 pM only slightly. Why should there be a high concentration of FVIIa in the assay? Should it come from the EVs? I cannot find any references describing this. FVIIai will bind to TF and therefore inhibit this activity, but will it block all other reactions for the alleged FVIIa? I cannot find documentation for this. When we get a large difference between samples with and without aTF, it is most likely due to a TF activity

The authors are focusing on the detection of very low concentrations of TF in plasma to be used for analyzing samples from venous thrombosis patients. In my opinion, procoagulant EVs from activated cells expressing large amounts of phosphatidylserine, play a more important role in thrombin generation in venous thrombosis than the traces of TF detected. Only EVs from plasma of certain cancer patients may have TF levels that can be associated with their events of thrombosis.

It is not correct that we focus on very low concentrations of TF. We agree that it is mainly interesting in certain cancer patients which we intend to investigate (which is also stated in the Introduction). That is the reason why we have established this method which we find more easy to use than the Mackman method.

We have now expressed that more clearly in the paper

Some minor points with strange sentences:
Line 127: “In seven normal volunteers TF activity was measured in plasma and in plasma from LPS-stimulated blood”. How do you define a normal volunteer? TF activity was not measured in plasma but in EVs isolated from plasma.

R: We have changed it to healthy volunteers. We have changed plasma to EVs.

Line 208: The levels in 7 normal persons.

We have changed it to healthy controls

Line 214: “marked samples with a low TF activity which were concentrated quite much (i.e. a high amount of EVs in”

It has been rephrased

Line 221: “without aTF, so this non-TF activity did not interfere much with the result”

We have changed non-TF activity to TF-independent activity

7. PLOS authors have the option to publish the peer review history of their article ([what does this mean?](https://eur03.safelinks.protection.outlook.com/?url=https%3A%2F%2Fjournals.plos.org%2Fplosone%2Fs%2Feditorial-and-peer-review-process%23loc-peer-review-history&data=05%7C01%7C%7C4659163a30064e9c739008db71267b87%7C5968b90c51a64f088b4750ffffbe2e4f%7C0%7C0%7C638228182329558832%7CUnknown%7CTWFpbGZsb3d8eyJWIjoiMC4wLjAwMDAiLCJQIjoiV2luMzIiLCJBTiI6Ik1haWwiLCJXVCI6Mn0%3D%7C3000%7C%7C%7C&sdata=JobrW6rhw413snOdZwbHgkcjVNAqvQgpfPDb%2Bp1Laaw%3D&reserved=0)). If published, this will include your full peer review and any attached files.


If you choose “no”, your identity will remain anonymous but your review may still be made public.


**Do you want your identity to be public for this peer review?** For information about this choice, including consent withdrawal, please see our [Privacy Policy](https://eur03.safelinks.protection.outlook.com/?url=https%3A%2F%2Fwww.plos.org%2Fprivacy-policy&data=05%7C01%7C%7C4659163a30064e9c739008db71267b87%7C5968b90c51a64f088b4750ffffbe2e4f%7C0%7C0%7C638228182329715060%7CUnknown%7CTWFpbGZsb3d8eyJWIjoiMC4wLjAwMDAiLCJQIjoiV2luMzIiLCJBTiI6Ik1haWwiLCJXVCI6Mn0%3D%7C3000%7C%7C%7C&sdata=%2B7sKBGPluTPvo00SBIHMmAIYzAuhv2oT7D0PYZmMTkE%3D&reserved=0).

Reviewer #1: **Yes:** Andrew D Meyer, MD, MS

Reviewer #3: No

[NOTE: If reviewer comments were submitted as an attachment file, they will be attached to this email and accessible via the submission site. Please log into your account, locate the manuscript record, and check for the action link "View Attachments". If this link does not appear, there are no attachment files.]

While revising your submission, please upload your figure files to the Preflight Analysis and Conversion Engine (PACE) digital diagnostic tool, [https://pacev2.apexcovantage.com/](https://eur03.safelinks.protection.outlook.com/?url=https%3A%2F%2Fpacev2.apexcovantage.com%2F&data=05%7C01%7C%7C4659163a30064e9c739008db71267b87%7C5968b90c51a64f088b4750ffffbe2e4f%7C0%7C0%7C638228182329715060%7CUnknown%7CTWFpbGZsb3d8eyJWIjoiMC4wLjAwMDAiLCJQIjoiV2luMzIiLCJBTiI6Ik1haWwiLCJXVCI6Mn0%3D%7C3000%7C%7C%7C&sdata=JlLfdUBzZN%2Fg9NJ3k1O96grFNlBSBvDbTvoEwqpcVw4%3D&reserved=0). PACE helps ensure that figures meet PLOS requirements. To use PACE, you must first register as a user. Registration is free. Then, login and navigate to the UPLOAD tab, where you will find detailed instructions on how to use the tool. If you encounter any issues or have any questions when using PACE, please email PLOS at [figures@plos.org](mailto:figures@plos.org). Please note that Supporting Information files do not need this step.
